# Nanoarchitectonics of Lotus Seed Derived Nanoporous Carbon Materials for Supercapacitor Applications

**DOI:** 10.3390/ma13235434

**Published:** 2020-11-29

**Authors:** Ram Lal Shrestha, Rashma Chaudhary, Timila Shrestha, Birendra Man Tamrakar, Rekha Goswami Shrestha, Subrata Maji, Jonathan P. Hill, Katsuhiko Ariga, Lok Kumar Shrestha

**Affiliations:** 1 Amrit Campus, Tribhuvan University, Kathmandu 44613, Nepal; swagatstha@gmail.com (R.L.S.); chaudharyreshma896@gmail.com (R.C.); timilastha@gmail.com (T.S.); 2 Tri-Chandra Multiple Campus, Tribhuvan University, Kathmandu 44600, Nepal; tamrakar_birendra@hotmail.com; 3 International Center for Materials Nanoarchitectonics (WPI-MANA), National Institute for Materials Science (NIMS), 1-1 Namiki, Ibaraki, Tsukuba 305-0044, Japan; GOSWAMI.Rekha@nims.go.jp (R.G.S.); MAJI.Subrata@nims.go.jp (S.M.); Jonathan.HILL@nims.go.jp (J.P.H.); 4 Graduate School of Frontier Sciences, The University of Tokyo, 5-1-5 Kashiwanoha, Kashiwa, Chiba 277-8561, Japan

**Keywords:** lotus seed, activated nanoporous carbons, energy storage, specific capacitance, supercapacitors

## Abstract

Of the available environmentally friendly energy storage devices, supercapacitors are the most promising because of their high energy density, ultra-fast charging-discharging rate, outstanding cycle life, cost-effectiveness, and safety. In this work, nanoporous carbon materials were prepared by applying zinc chloride activation of lotus seed powder from 600 °C to 1000 °C and the electrochemical energy storage (supercapacitance) of the resulting materials in aqueous electrolyte (1M H_2_SO_4_) are reported. Lotus seed-derived activated carbon materials display hierarchically porous structures comprised of micropore and mesopore architectures, and exhibited excellent supercapacitance performances. The specific surface areas and pore volumes were found in the ranges 1103.0–1316.7 m^2^ g^−1^ and 0.741–0.887 cm^3^ g^−1^, respectively. The specific capacitance of the optimum sample was ca. 317.5 F g^−1^ at 5 mV s^−1^ and 272.9 F g^−1^ at 1 A g^−1^ accompanied by high capacitance retention of 70.49% at a high potential sweep rate of 500 mV s^−1^. The electrode also showed good rate capability of 52.1% upon increasing current density from 1 to 50 A g^−1^ with exceptional cyclic stability of 99.2% after 10,000 cycles demonstrating the excellent prospects for agricultural waste stuffs, such as lotus seed, in the production of the high performance porous carbon materials required for supercapacitor applications.

## 1. Introduction

Most lignocellulose-based materials can be used as precursors for the production of nanoporous activated carbon materials [1]. Activated carbon materials are some of the most versatile and commonly used adsorbents due to their exceptionally high surface areas and micropore volumes [2,3,4,5,6,7], extensive adsorption capabilities, fast adsorption kinetics, and relative ease of regeneration [8]. Currently, chemically activated carbon materials are widely used as high-performance electrochemical double-layer capacitors (EDLCs) or supercapacitor electrodes [9]. EDLCs present improved cycling stabilities and power densities over conventional rechargeable batteries and supercapacitors are a promising technology for addressing peak power demands for light electronic applications, including mobile devices and electric vehicles [10]. EDLCs can also be used as supporting sources of power for the startup and speeding up the electric cars and other high-performance automobiles [11]. However, supercapacitors are subject to significant disadvantages due to their comparatively low specific energy (energy density) (1–10 Wh kg^−1^) in comparison to lead-acid batteries (30–40 Wh kg^−1^) and lithium-ion batteries (160 Wh kg^−1^) [12,13,14]. For this reason, intense investigations have been conducted to improve the performance of supercapacitors with respect to the energy density [15,16,17,18,19,20]. Two methods commonly used to achieve this are widening of the operating potential window or increasing the specific capacitance of the electrode materials. More effort has been made to the latter of these since widening of the potential window is subject to some limitations [21]. Previous research has revealed that electrode specific capacitance is directly correlated with porosity along with specific surface area, pore-volume, interconnection among pores, and conductivity [12,13,14,15,16,17,18,19,20,21,22,23,24]. Cost and eco-friendliness of the electrode materials are also major considerations [9,25,26,27,28].

In addition to the molecular nanocarbon materials—such as fullerene, graphene, and carbon nanotubes, porous carbon materials derived from biomass—have been comprehensively employed as the electrode materials in supercapacitor applications because of low-cost and extraordinary specific surface area and large porosity [2,8,9,29,30,31,32,33]. Activation of the precursor can be carried out either by physical or chemical means [34]. Chemical activation is usually performed using different acidic (e.g., H_3_PO_4_), basic (e.g., KOH), or neutral reagents (e.g., ZnCl_2_) [21,35]. In this study, lotus seed powder was chemically activated using ZnCl_2_. ZnCl_2_ is a well-known dehydrating agent and it promotes the breakdown of carbon materials during the carbonization process, and also controls the tar formation [36]. Chemical activation methods are cost-effective giving higher yields, better developed porous structures, and require lower temperatures than physical activation methods [37,38,39,40,41,42,43]. The preparation of nanoporous activated carbon materials usually involves two steps, namely carbonization of the precursor and chemical activation of the carbonized char over a wide temperature ranges from 400 °C to 1000 °C under a constant flow of argon or nitrogen gas. Non-carbonaceous light elements are generally eliminated during the carbonization process [44] with the remaining carbon mostly forming irregular condensed aromatic systems with vacancies containing disordered amorphous carbon originating from the deposition and decomposition of tars [44,45]. When ZnCl_2_ is present during carbonization of lignocellulose, it undergoes pyrolytic decomposition and enrichment of porosity due to depolymerization and dehydration of the resulting biochar [46].

In this contribution, we report the electrochemical energy storage supercapacitance performances of ZnCl_2_ activated hierarchically porous carbon materials derived from lotus seed in aqueous 1 M sulfuric acid solution at 25 °C. Biochar was initially prepared by the heat-treatment of lotus seed powder in air at 300 °C, with the product then being chemically activated by its impregnation with ZnCl_2_ at a weight ratio of 1:1. Subsequent carbonization was carried out at higher temperatures (600–1000 °C). The morphology, structure, and textural properties of the prepared carbon materials were examined by scanning electron microscopy, powder X-ray diffraction, Raman scattering, and analysis of their nitrogen adsorption isotherms.

## 2. Materials and Methods

### 2.1. Fabrication of Hierarchically Porous Carbons Materials

Lotus seed (Plant scientific name: *Nelumbo nucifera* (Gaertn), a perennial aquatic plant) was collected from a local herbarium market at Kathmandu, Nepal. The Lotus seed was dried at 120 °C in an oven for 10 h and crushed in an electric grinder. The crushed powder was then heat-treated at 300 °C for 3 h in the air, and biochar was obtained. This was followed by mixing with ZnCl_2_ (1:1 weight ratio), and final carbonizations were carried out at high temperatures (600–1000 °C) under a flow of nitrogen gas (120 cm^3^ min^−1^) in a tube furnace (KOYO, Tokyo, Japan) at a heating speed of 5 °C min^−1^ and a hold time of 3 h. The resulting carbon materials were washed with dilute aqueous hydrochloric acid (0.5 M) to remove unreacted ZnCl_2_ then also washed with distilled water multiple times until the pH of the washings was neutral. The final products were vacuum dried at 80 °C for 6 h then ground finely to powders. The chemically activated nanoporous carbon samples are denoted as LTSC_Z600, LTSC_Z800, and LTSC_Z1000 based on the carbonization temperature. A control material was prepared by directly carbonizing (without ZnCl_2_ activation) Lotus seed powder at 800 °C with the product obtained being denoted as LTS_800.

### 2.2. Characterizations

Nanoporous carbon materials obtained from the ZnCl_2_ activation of Lotus seed were characterized by scanning electron microscopy (SEM: S-4800, Hitachi Co. Ltd. Tokyo, Japan operated at 10 kV), powder X-ray diffraction (XRD: Rigaku X-ray diffractometer, RINT, Tokyo, Japan operated at 40 kV), Raman scattering (NRS-3100, JASCO, Tokyo, Japan: green laser of wavelength 524.5 nm at 0.01 mW power was used), Fourier-transform infrared spectroscopy (FTIR: Nicolet 4700, Thermo Electron Corporation, Waltham, MA, USA), and nitrogen adsorption isotherm analysis using an automatic adsorption instrument (Quanta chrome Autosorb-iQ2, Boynton Beach, FL, USA) at liquid nitrogen temperature (−196.1 °C). Barrett–Joyner–Halenda (BJH) method and density functional theory (DFT) were used to obtain pore size distributions. Note that BJH and DFT theory assumes cylindrical and slit-like pores, respectively.

### 2.3. Electrode Preparation and Electrochemical Studies

The electrochemical performances (specific capacitance, rate capability, and cycle life) of Lotus seed-derived nanoporous carbon materials was calculated based on cyclic voltammetry (CV) and galvanostatic charge–discharge (CD) measurements. The electrochemical measurements were performed using a three-electrode system in 1 M H_2_SO_4_ aqueous electrolyte solution at 25 °C. Modified glassy carbon electrode (GCE, ALS Co., Ltd. Tokyo, Japan) with the material for analysis was used as the working electrode and Ag/AgCl and a platinum wire were used as counter and reference electrodes, respectively. Working electrodes were prepared as follows. First, the GCE was cleaned and mirror polished with a slurry of alumina (Al_2_O_3_), cleaned with distilled water, and vacuum dried at 60 °C for 3 h. Separately, the carbon material for analysis was dispersed in a solvent mixture (ethanol-water = 1:4 v/v mixture: concentration of 1 mg mL^−1^) and sonicated in a bath sonicator (BRANSON 3510, Branson, Hampton, NH, USA) for 1 h. The obtained suspension (3 μL) was dropped onto the GCE surface and dried at 60 °C for 6 h for removal of the solvent. A Nafion solution (5 μL: 5% in ethanol) was added on top of the active materials as a binder and again by dried at 60 °C for 12 h. Electrochemical impedance spectroscopy (EIS) measurements were conducted in 1 M H_2_SO_4_ electrolyte at an open-circuit potential. The frequency range was varied from 0.01 Hz to 100 kHz at an amplitude of 5 mV. CV, CD, and EIS measurements were performed on a CHI 660E workstation (CH Instruments, Inc. Austin, TX, USA) at 25 °C.

From the CV curves, specific capacitance (*C*_s_) was calculated as

(1) $$C_{s}=\frac{1}{v.\;m.\;\Delta V}\int IdV$$

where *I*, *ν*, *m*, and ∆*V,* respectively denote the current (A), scan rate (V s^−1^), the mass of active electrode materials (g), and the potential window (V).

Similarly, from CD curves, *C*_s_ was calculated as

(2) $$C_{s}=\frac{I\cdot t}{m\cdot \Delta V}$$

where *I, t, m,* and ∆*V* represent the discharge current (A), discharge time (s), mass of active electrode materials (g), and the operating potential window (*V*_final_–*V*_initial_), respectively.

## 3. Results and Discussion

**Figure 1:**
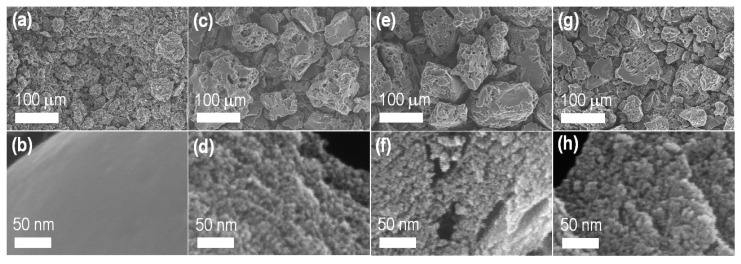
SEM observations of nanoporous activated carbon materials derived from lotus seed: (**a,b**) LTS_800; (**c,d**) LTSC_Z600; (**e,f**) LTSC_Z800; (**g,h**) LTSC_Z1000.

SEM imaging revealed the porous structure of the carbon materials derived from lotus seed. Figure 1 shows typical SEM images of LTS_800 (the directly carbonized sample without ZnCl_2_: Figure 1a,b) and ZnCl_2_ activated carbon materials (LTSC_Z600: Figure 1c,d; LTSC_Z800: Figure 1e,f; and LTSC_Z1000: Figure 1g,h). Additional SEM images are supplied in the Supplementary Materials (LTS_800: Figure S1; LTSC_Z600: Figure S2; LTSC_Z800: Figure S3; and LTSC_Z1000: Figure S4).

Low magnification SEM images reveal irregular morphology and dimensions (<200 μm) of the carbon granules with porous surface structures (Figure 1a,c,e,g). Surface pore structure (micro- and mesopores) is lacking in the directly carbonized sample (Figure 1a,b) while, in the ZnCl_2_ activated samples (Figure 1d,f,h), pore structures are present demonstrating the key role of the dehydrating agent. Apart from the generation of micro- and mesopore structures, ZnCl_2_ activation also led to macroporous channels on the rough surface of the carbon particles (Figure 1c,e,g), a feature commonly observed in chemically activated carbons [47,48,49,50,51]. Because of the well-developed porosity, ZnCl_2_ activated lotus seed carbon materials are expected to be useful in high-level electrochemical energy storage applications.

Oxygenated surface functional groups are generally observed in biomass-derived activated carbons [52,53]. Surface functional groups of nanoporous carbons from lotus seed were detected in their FTIR spectra. The spectra (Figure S5) contain a C–O stretching absorption at 1087 cm^−1^ and a weak peak at 1631 cm^−1^ due to aromatic C=C stretching vibrations commonly observed in the activated carbons [53]. Although it is not clear, a weak band at 2925–2972 cm^−1^ indicates C–H stretching indicating the presence of an aromatic ring. The broad vibration at 3446 cm^−1^ is due to OH (str) suggesting the presence of an alcoholic functional group or from moisture [53].

**Figure 2:**
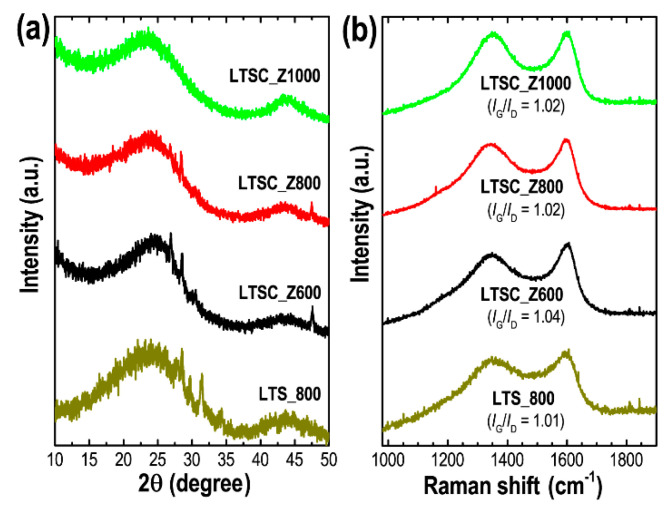
(**a**) XRD patterns, and (**b**) Raman scattering spectra of LTS_800, LTSC_Z600, LTSC_Z800, and LTSC_Z1000.

Structural properties of the Lotus seed-derived nanoporous carbon materials were further characterized by XRD and Raman scattering spectroscopy. Figure 2a shows the XRD patterns of these carbon materials. Diffraction patterns indicate an amorphous structure with XRD peaks at angles of ~23° and 43°, which are related to (002) and (100) diffraction planes of graphitic carbons characteristic of nanoporous activated materials derived from biomass or lignocellulosic precursors [54]. Although carbonizations were carried out at high temperature up to 1000 °C, the observed carbon materials have poorly ordered structure with only partial graphitization. A high degree of graphitization is expected at higher carbonization temperature (>1000 °C). Small peaks observed particularly in the LTS_800 and LTSC_Z600 samples are due to impurities.

Raman scattering spectra (Figure 2b) further confirm the amorphous state of these carbon materials. Two characteristic Raman bands at ~1350 (*D* band) and ~1588 cm^−1^ (*G* band) can be seen in all the samples, which are typical features of the activated amorphous carbon materials. The *D* band reveals the disordered structure of carbon induced by impurities present during high-temperature carbonization, whereas the *G* band reflects an ordered graphitic layer structure. To estimate the graphitization degree, the intensity ratio of *G* and *D* bands (*I*_G_/*I*_D_) was calculated from the obtained raw data. The obtained *I*_G_/*I*_D_ ratio in the range of 1.01 to 1.04 demonstrates amorphous graphitic carbon structure with fewer defects [55].

**Figure 3:**
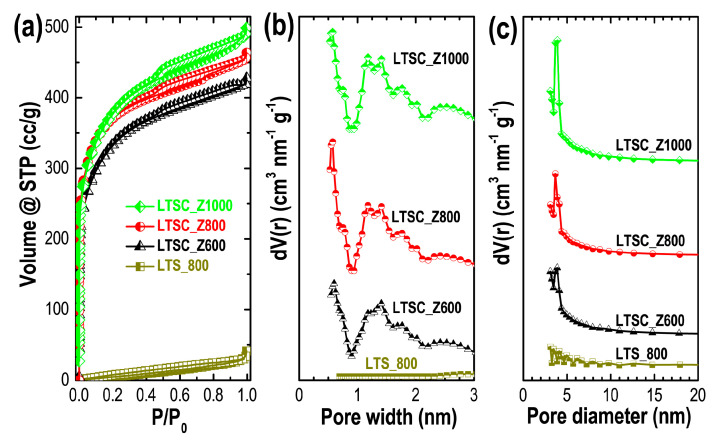
(**a**) Nitrogen sorption (adsorption/desorption) isotherms of LTS_800, LTSC_Z600, LTSC_Z800, and LTSC_Z1000, and the pore size distributions curves as obtained from (**b**) DFT method, and (**c**) BJH method.

Porosity properties (specific surface areas, pore volumes, average pore sizes, and pore size distributions) of the prepared carbon materials were evaluated by nitrogen adsorption measurements. Figure 3a shows the nitrogen adsorption/desorption isotherms of LTS_800, LTSC_Z600, LTSC_Z800, and LTSC_Z1000.

The nonporous structure of the LTS_800 (also found in its SEM images) results in a Type-III adsorption isotherm. Thermolysis of lotus seed powder at a high temperature (800 °C) without activating agent randomly disrupts the carbon skeleton leading to a macroporous or nonporous structure. In contrast, in case of ZnCl_2_-activation pyrolytic decomposition occurs and porosity enhancement is obtained due to depolymerization and dehydration of biochar. The well-developed porosities of the ZnCl_2_-activated samples can be judged from the mixed Type-I/Type-IV adsorption isotherms. A Type-I adsorption isotherm corresponds to a microporous structure, while a Type-IV isotherm corresponds to a mesoporous form indicating a hierarchically porous structure [56]. Nitrogen uptake of LTS_800 is deficient compared to the chemically activated carbon materials because of lack of porous structure. Increased adsorption of nitrogen by activated samples (LTSC_Z600, LTSC_Z800, and LTSC_Z1000) at a lower relative pressure (P/P_0_ < 0.03) reveals a microporous structure, which contributes greatly to enhance the specific surface area and charge storage capacity. Clear hysteresis loops caused by capillary condensation at higher relative pressures indicate a mesoporous structure, which promotes electrolyte ion diffusion in supercapacitors electrode materials. Figure 3b,c show respectively the pore size distribution curves as obtained from the DFT and BJH methods. The pore size distribution profiles clearly indicate well-defined micropores (Figure 3b) and mesopores (Figure 3c) and the presence of a hierarchical micro/mesopore structure in the activated samples, LTSC_Z600, LTSC_Z800, and LTSC_Z1000.

The porosity properties of ZnCl_2_-activated Lotus seed carbons are summarized in Table 1. The low surface area of the LTS_800 (46.1 m^2^ g^−1^) is due to its lack of porosity. In contrast, activated samples show very high surface areas with the optimal sample exhibiting a surface area of 1316.7 m^2^ g^−1^ and large pore volume of 0.794 cm^3^ g^−1^. The average mesopore diameter of the optimum sample was calculated to be 3.67 nm.

**Figure 4:**
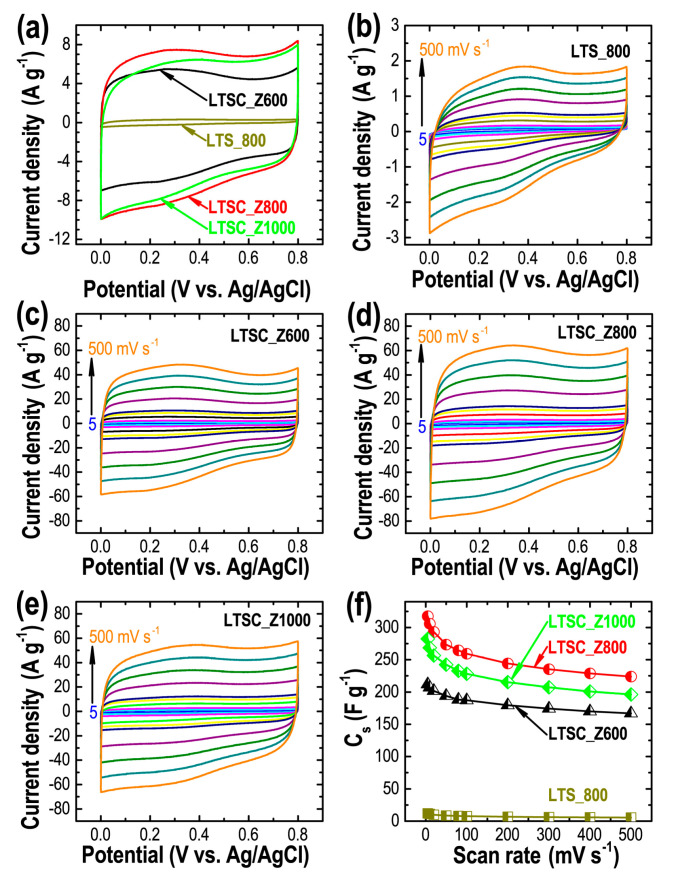
(**a**) CV curves of the carbon materials at a fixed scan rate of 50 mV s^−1^, and the corresponding CV profiles at different scan rates (5 to 500 mV s^−1^). (**b**) LTS_800, (**c**) LTSC_Z600, (**d**) LTSC_Z800, (**e**) LTSC_Z1000, and (**f**) *C*_s_ obtained from CV profiles for all the samples.

Inspired by the high specific surface area and well-developed porosity of the lotus seed-derived nanoporous carbon materials, the electrochemical energy storage capacities of the prepared materials were studied by CV and CD measurements. *C*_s_, rate capability, and cycle stability of the electrode materials were also calculated. Figure 4a displays the typical CV profiles of the carbon materials at a scan rate of 50 mVs^−1^ where the semi-rectangular shape of the CV curves signifies EDLC type behavior of the electrodes [57]. The total integral current output following the order LTSC_Z800 > LTSC_Z1000 > LTSC_Z600 > LTS_800 reveals the effect of porosity on the properties where the higher specific surface area leads to improved charge storage capacity (Table 1). The maximum current collection was observed for LTSC_Z800. The formation of sufficient micropores in the activated carbon samples is advantageous as it can accommodate more electrolyte ions forming the electrical double-layers [58]. Simultaneously, the development of mesopores promotes the diffusion of electrolyte ions to the electrode surface [59]. CV profiles of all the samples (LTS_800: Figure 4b, LTSC_Z600: Figure 4c, LTSC_Z800: Figure 4d, and LTSC_Z1000: Figure 4e) show that the total integral current increases with scan rate and the quasi-rectangular shape of the curves is retained even at a high scan rate of 500 mV s^−1^, which explains the rapid charge propagation with excellent reversibility at high scan rate. Using Equation (1) *C*_s_ were calculated from the CV curves (Figure 4f). As expected, the carbon sample obtained without ZnCl_2_ showed poor energy storage capacity because of the low surface area and poor porosity. The LTS_800 electrode showed a low specific capacitance of 11.9 F g^−1^ at 5 mV s^−1^. On the other hand, ZnCl_2_-activated samples exhibited enhanced supercapacitances. The best sample (LTSC_Z800) attained the highest capacity of 317.5 F g^−1^ at a low scan rate of 5 mV s^−1^ and retained 70.4% capacitance at a high scan rate of 500 mV s^−1^. *C*_s_ of samples LTSC_Z600 and LTSC_Z1000 were calculated to be 212.0 and 282.4 F g^−1^, respectively.

**Figure 5:**
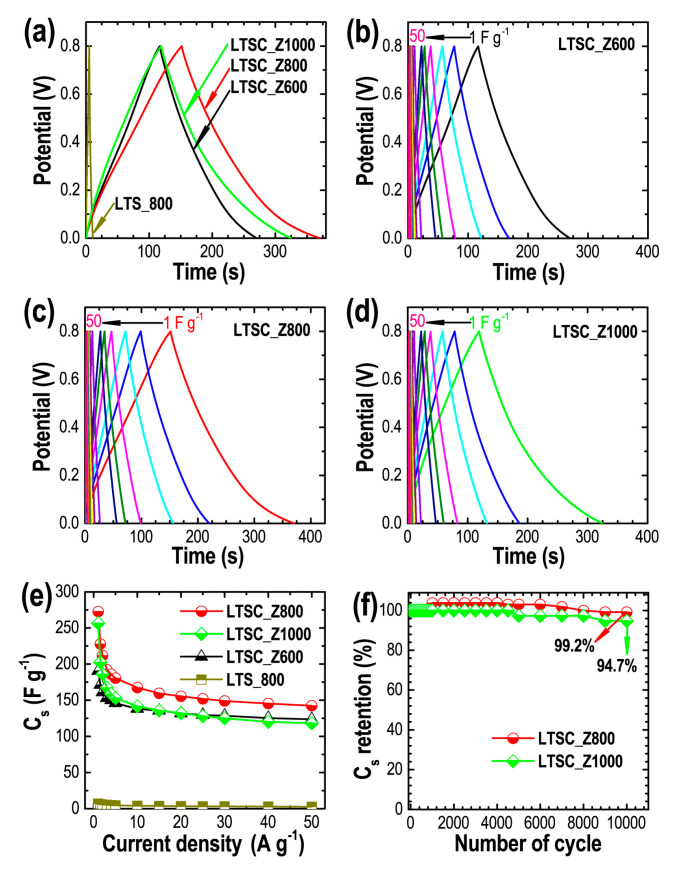
(**a**) CD profiles of the carbon materials at 1 A g^−1^, and CD profiles vs. current densities (1–50 A g^−1^), (**b**) LTSC_Z600, (**c**) LTSC_Z800, and (**d**) LTSC_Z1000, as typical examples, (**e**) *C*_s_ vs. current density, and (**g**) cyclic stability of the LTSC_Z800 and LTSC_Z1000 electrodes for 10,000 charging–discharging cycles as typical example.

Figure 5a compares the CD profiles of all the samples at 1 A g^−1^. All the samples exhibit semi-triangular shape CD profiles inferring EDLC behavior of the materials [27,60,61] with well-balanced charge storage that can be judged from the linear decay of the discharge curves [62]. LTS_800 showed the shortest discharge time, illustrating less effective charge storage while the LTSC_Z800 showed the extended discharge time having the highest energy storage capacity of the samples studied. CD profiles vs. current densities (1–50 A g^−1^) are shown for LTSC_Z600, LTSC_Z800, and LTSC_Z1000 (Figure 5b–d). Specific capacitances were calculated (using Equation (2)) as being 7.1 F g^−1^ (LTS_800), 190.6 F g^−1^ (LTSC_Z600), 272.9 (LTSC_Z800), and 256.4 (LTSC_Z1000) at 1 A g^−1^. Figure 5e shows the *C*_s_ vs. current density curves for these carbon materials. Because of the high surface area (mainly micropore surface area) the LTSC_Z800 showed the highest electrochemical energy storage performance and also a good rate capability of 52.3% at 50 A^−1^.

Cycle life of supercapacitor devices is a key parameter in real world applications. We have examined the cyclic stability performance of selected electrodes up to 10,000 charging–discharging cycles (Figure 5f). Both electrodes studied exhibited exceptional cycle lifetimes. LTSC_Z800 electrode retained 99.2% capacity while LTSC_Z1000 electrode retained 94.7% capacity demonstrating the potential applicability of our materials in high performance electrochemical double-layer capacitors.

We have also compared the *C*_s_ of the ZnCl_2_-activated Lotus seed nanoporous carbon electrodes with similar other biomass-derived nanoporous carbon materials (Table 2).

**Figure 6:**
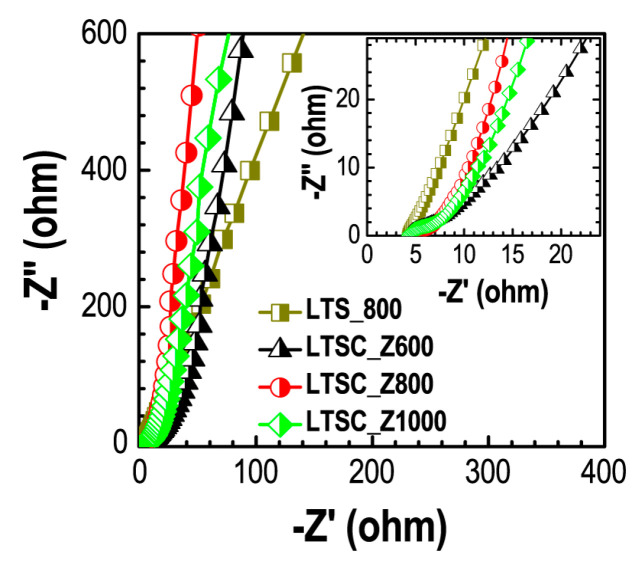
Nyquist plots of the prepared carbon materials (LTS_800, LTSC_Z600, LTSC_Z800, and LTSC_Z1000) obtained from EIS measurements. Inset shows the magnified plots.

Figure 6 shows Nyquist plots obtained from the EIS measurements. The poor semicircular behavior in the high frequency region indicates the ideal EDLC behavior of the electrode materials.

Fast electrolyte ion transport in the activated samples (LTSC_Z600, LTSC_Z800, and LTSC_Z1000) can be judged from the sharp rise of the curves at the lower frequency region of Nyquist plots. Hierarchically porous structure comprising of both the mesopore and micropore architectures enables fast electrolyte ion transfer within the electrode materials and thus contributes to enhancing the supercapacitance performance. Whereas, poor ion transport in the LTS_800 is caused due to the absence of well-developed porosity. From Nyquist plots the equivalent series resistance (ESR) was ca. 4.16 Ω (LTS_800), 4.17 Ω (LTSC_Z600), 4.37 Ω (LTSC_Z800), and 4.32 Ω (LTSC_Z1000). The ESR values of all the samples are comparable demonstrating that the difference in the specific surface area and pore volume of the materials are mainly responsible for the better supercapacitance performance of the LTSC_Z800.

It should be noted that the high surface area, well-developed porosity with large pore volume, narrow and well-defined pore size distributions, and pore network connectivity in porous carbon materials comprising of macro, meso, and micropores architectures are the key parameters in supercapacitors applications of carbon materials [65]. Using conventional templating methods only limited examples have been realized in the synthesis of hierarchically porous carbons. On the other hand, non-renewable carbon sources such as inorganic precursors have been utilized for the preparation of high-performance porous carbon materials in energy storage, sensing, and catalysis applications [66]. However, from a long-term perspective and for sustainability, it is essential to look for bio- and renewable carbon sources for the low-cost and scalable production of porous carbon materials that can retain interconnected porous framework networks and exhibit high specific surface areas with required pore sizes and structures [67]. Needless to say, graphene, carbon nanotubes, and carbide-derived carbons have been comprehensively studied as electrode materials in supercapacitor applications, efforts are being made to enhance their electrochemical energy storage performances. Graphene suffers from inherent restacking of layers, and due to the lack of well-developed porosity, network pores structures, and hierarchy in the pore architectures, these carbon materials needs post-treatments or optimization of specific surface and adjusting the distribution of pores so that more electroactive sites can be generated for the free electrolyte ion transport pathways thereby improving the specific capacitance and overall rate capability. However, to fabricate such electrode materials, unsustainable and expensive starting raw materials are generally used. Therefore, low-cost and sustainable biomass and agro-waste are considered as alternative carbon sources. Owing to compositional and morphological variations, intrinsic pore architectures, and renewability lignocellulose-derived carbon materials have been greatly explored to fabricate electrodes for advanced supercapacitors. Compared to the porous carbons obtained from inorganic precursors, biomass-derived carbons offer high specific surface areas and large porosity, intrinsic doping of different heteroatoms, and good electrical conductivity. Furthermore, specific surface area and porosity properties of biomass-derived carbons can be tuned by a subtle balance of synthetic conditions such as temperature, type of activating agents, impregnation ratio of activating agents, etc. Thus, judging from the surface morphology, structure, porosity properties, and electrochemical performances, it can be inferred that this work provides a simple, low-cost, and scalable method of producing high-performance nanoporous carbon materials from the lignocellulosic precursor, which would have potential as the electrode materials in supercapacitor applications.

## 4. Conclusions

In summary, the electrochemical energy storage performances of hierarchically porous carbon materials produced by ZnCl_2_-activation of lotus seed powder are reported in an aqueous electrolyte in a three-electrode system. ZnCl_2_ activated lotus seed carbon materials obtained by the high-temperature carbonizations (600–1000 °C) exhibit a hierarchically porous structure comprising a micro-and mesopore architecture. Cyclic voltammetry and chronopotentiometry results indicate the excellent energy storage performances of the materials. The optimal sample exhibited a high specific capacitance of 317.5 F g^−1^ at a scan rate of 5 mV s^−1^ and 272.9 F g^−1^ at a current density of 1 A g^−1^ accompanied by high capacitance retention of 70.49% at a very high scan rate of 500 mV s^−1^. The electrode also had excellent rate capability, retaining 52.1% capacity at a high current density of 50 A g^−1^ accompanied by exceptionally high cyclic stability (99.2%) after 10,000 charging–discharging cycles. The obtained excellent electrochemical performances of the materials can be attributed to the high surface area (1316.7 m^2^ g^−1^) with associated large pore volumes (0.887 cm^3^ g^−1^). Judging from the surface textural properties (surface areas and porosity) and electrochemical performance, it can be concluded that lotus seed, an agricultural waste product (agro-waste), is a good precursor material for the scalable production of high-performance supercapacitor electrode materials.

## Figures and Tables

**Table 1:** Porosity properties of ZnCl_2_ activated lotus seed carbon materials as obtained from DFT and BJH methods

Carbon Sample	*SSA* (m^2^ g^−1^)	*S*_micro_ (m^2^ g^−1^)	*S*_meso_ (m^2^ g^−1^)	*V*_p_ (cm^3^ g^−1^)	*V*_micro_ (cm^3^ g^−1^)	*D*_meso_ (nm)
LTS_800	46.1	18.8	27.3	0.102	0.044	3.09
LTSC_Z600	1103.0	996.7	106.3	0.741	0.592	3.88
LTSC_Z800	1316.7	1213.6	103.1	0.794	0.642	3.67
LTSC_Z1000	1297.6	1158.5	139.1	0.887	0.690	3.89

*SSA* = specific surface area, *S*_micro_ = micropore surface area, *S*_meso_ =mesopore surface area, *V*_p_ = total pore volume, *V*_micro_ = micropore volume obtained from the DFT method, and *D*_meso_ = average mesopore diameter obtained by the BJH method.

**Table 2:** Specific capacitances of biomass-derived nanoporous activated carbon electrodes including from lotus seed-derived carbon electrodes (this work)

Biomass	Electrolyte	Current Density/Scan Rate	Specific Capacitance (F g^−1^)	Reference
Lotus seed	1 M H_2_SO_4_	1 A g^−1^/5 mV s^−1^	272.9/317.5	This work
Lapsi seed	1 M H_2_SO_4_	1 A g^−1^	284	[36]
Jackfruit seed	1 M H_2_SO_4_	1 A g^−1^	261.3	[37]
Bamboo	1 M H_2_SO_4_	5 mV s^−1^	256	[38]
Washnut	1 M H_2_SO_4_	1 A g^−1^	225.1	[46]
Cotton	3 M KOH	0.3 A g^−1^	221.7	[63]
Corn cob	0.5 M H_2_SO_4_	0.5 A g^−1^	210	[53]
Beech (Fagus sylvatica)	1 M KOH	20 mA g^−1^	133	[64]

## Supplementary Materials

The following are available online at https://www.mdpi.com/1996-1944/13/23/5434/s1, Figure S1: Additional SEM images of the directly carbonized Lotus seed carbons (LTS_800); Figure S2: Additional SEM images of ZnCl_2_ activated Lotus seed carbons, LTSC_Z600; Figure S3: Additional SEM images of ZnCl_2_ activated Lotus seed carbons, LTSC_Z800; Figure S4: Additional SEM images of ZnCl_2_ activated Lotus seed carbons, LTSC_Z1000; Figure S5: FTIR spectra of Lotus seed-derived carbons LTS_800, LTSC_Z600, LTSC_Z800, and LTSC_Z1000 recorded at 25 °C.
