# Supplementary material for: Nanoarchitectonics of Lotus Seed Derived Nanoporous Carbon Materials for Supercapacitor Applications
Source: Materials (Basel). 2020 Nov 29;13(23):5434. doi: 10.3390/ma13235434 (PMC7730822; doi:10.3390/ma13235434)
Supplement: Supplementary file 1 [file materials-13-05434-s001.pdf]

materials

Supplementary Information

Nanoarchitectonics of Lotus Seed Derived Nanoporous Carbon Materials for Supercapacitor Applications

Ram Lal Shrestha [1], Rashma Chaudhary [1], Timila Shrestha [1], Birendra Man Tamrakar [2], Rekha Goswami Shrestha [3], Subrata Maji [3], Jonathan P. Hill [3], Katsuhiko Ariga [3,4,*], and Lok Kumar Shrestha [3,*]

1 Amrit Campus, Tribhuvan University, Kathmandu 44613, Nepal; swagatstha@gmail.com (R.L.S.); chaudharyreshma896@gmail.com (R.C.); timilastha@gmail.com (T.S.)
2 Tri-Chandra Multiple Campus, Tribhuvan University, Kathmandu 44600, Nepal; tamrakar_birendra@hotmail.com
3 International Center for Materials Nanoarchitectonics (WPI-MANA), National Institute for Materials Science (NIMS), 1-1 Namiki, Ibaraki 305-0044 Tsukuba Japan; GOSWAMI.Rekha@nims.go.jp (R.G.S.); MAJI.Subrata@nims.go.jp (S.M.); Jonathan.HILL@nims.go.jp (J.P.H.)
4 Graduate School of Frontier Sciences, The University of Tokyo, 5-1-5 Kashiwanoha, Kashiwa, Chiba 277-8561, Japan
* Correspondence: ARIGA.Katsuhiko@nims.go.jp (K.A.); Tel.: +81-29-860-4597; SHRESTHA.Lokkumar@nims.go.jp (L.K.S.); Tel.: +81-29-860-4809;

Received: 9 October 2020; Accepted: 26 November 2020; Published: date

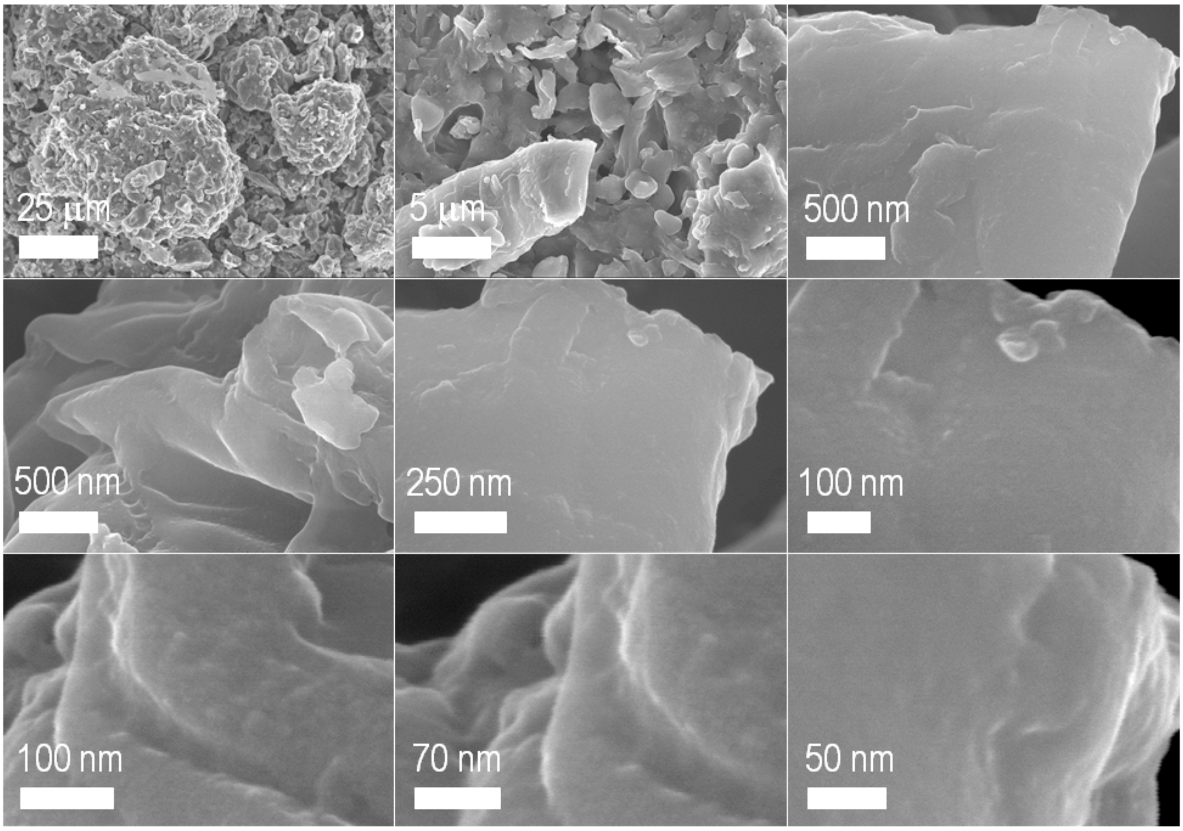

Figure S1. Additional SEM images of the directly carbonized Lotus seed carbons (LTS_800).

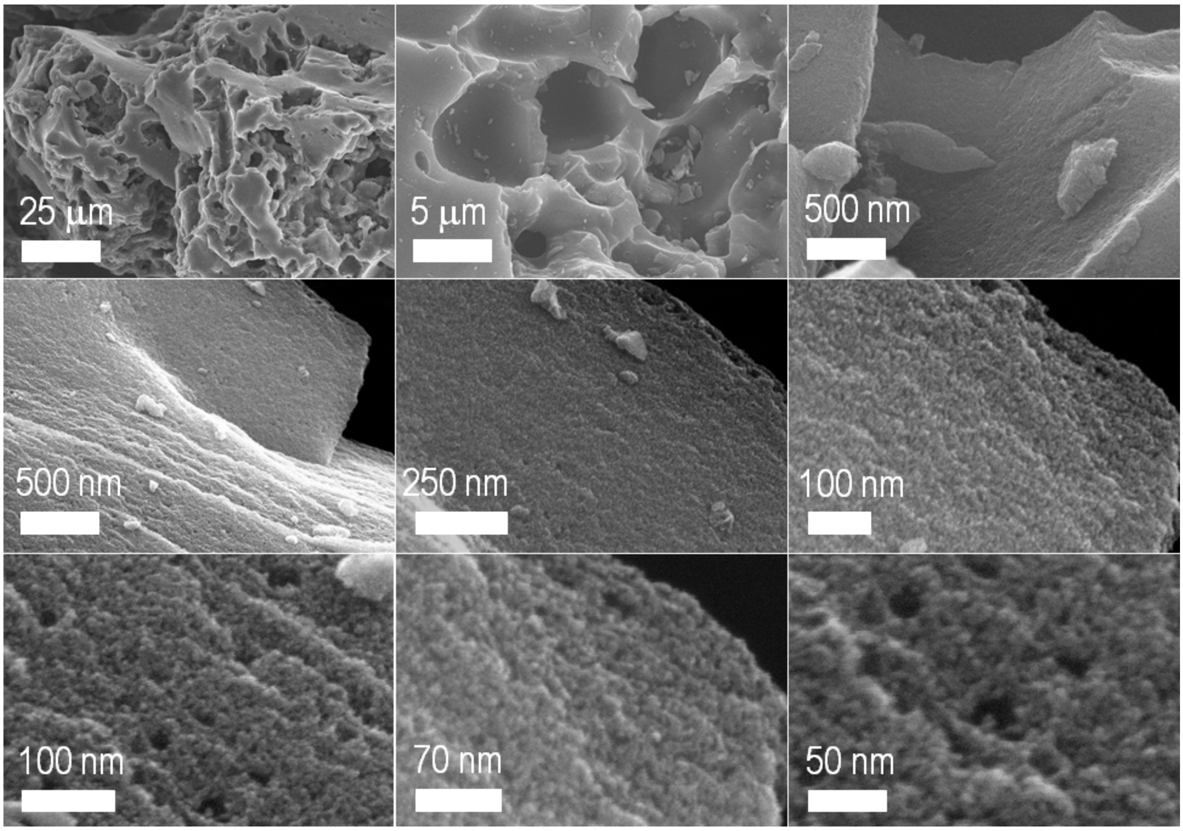

Figure S2. Additional SEM images of $ZnCl_2$ activated Lotus seed carbons, LTSC_Z600.

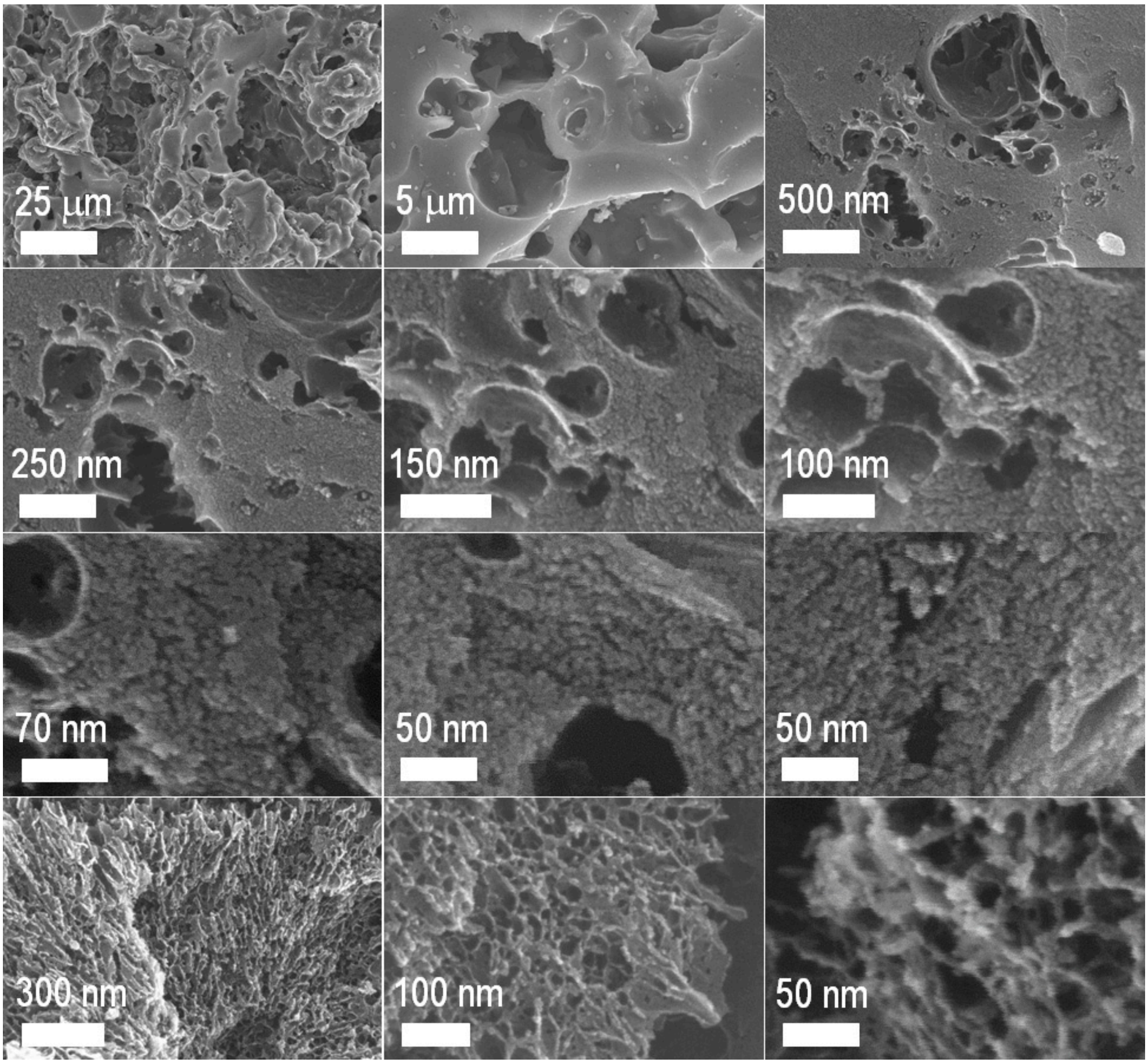

Figure S3. Additional SEM images of $ZnCl_2$ activated Lotus seed carbons, LTSC_Z800.

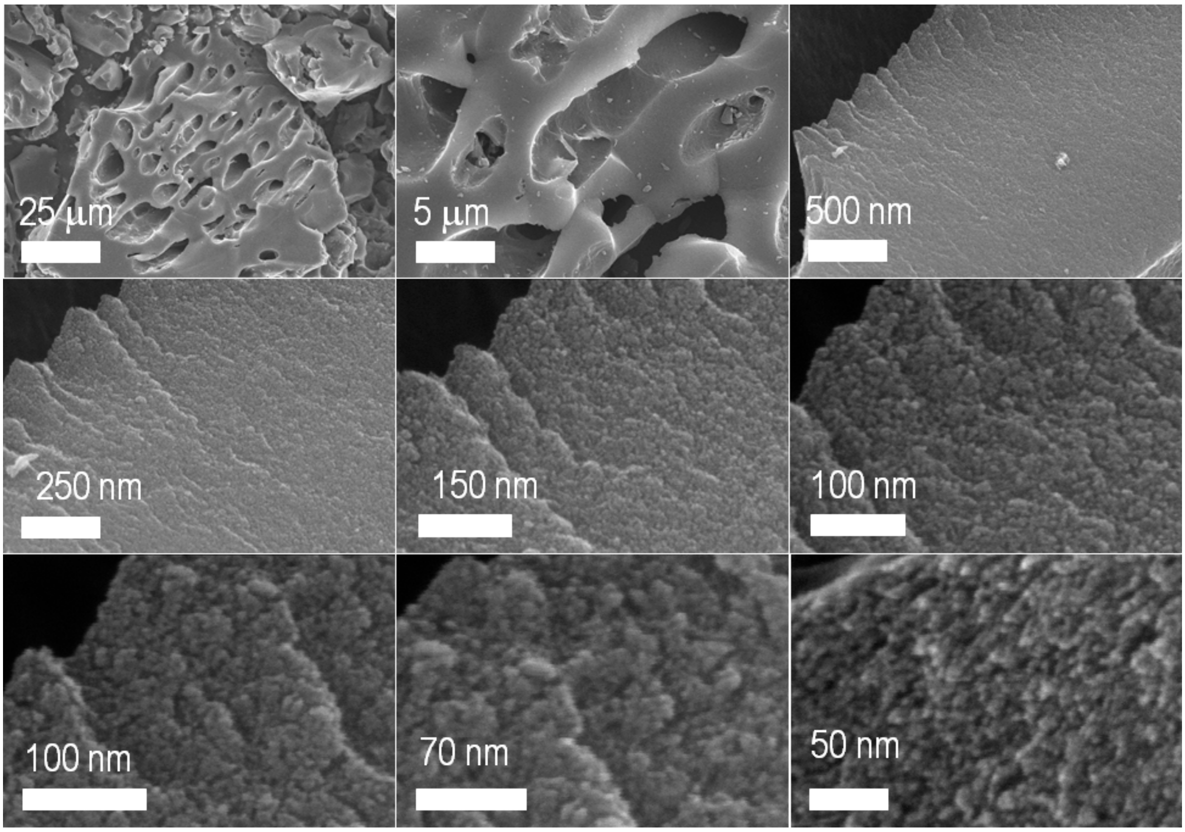

Figure S4. Additional SEM images of $ZnCl_2$ activated Lotus seed carbons, LTSC_Z1000.

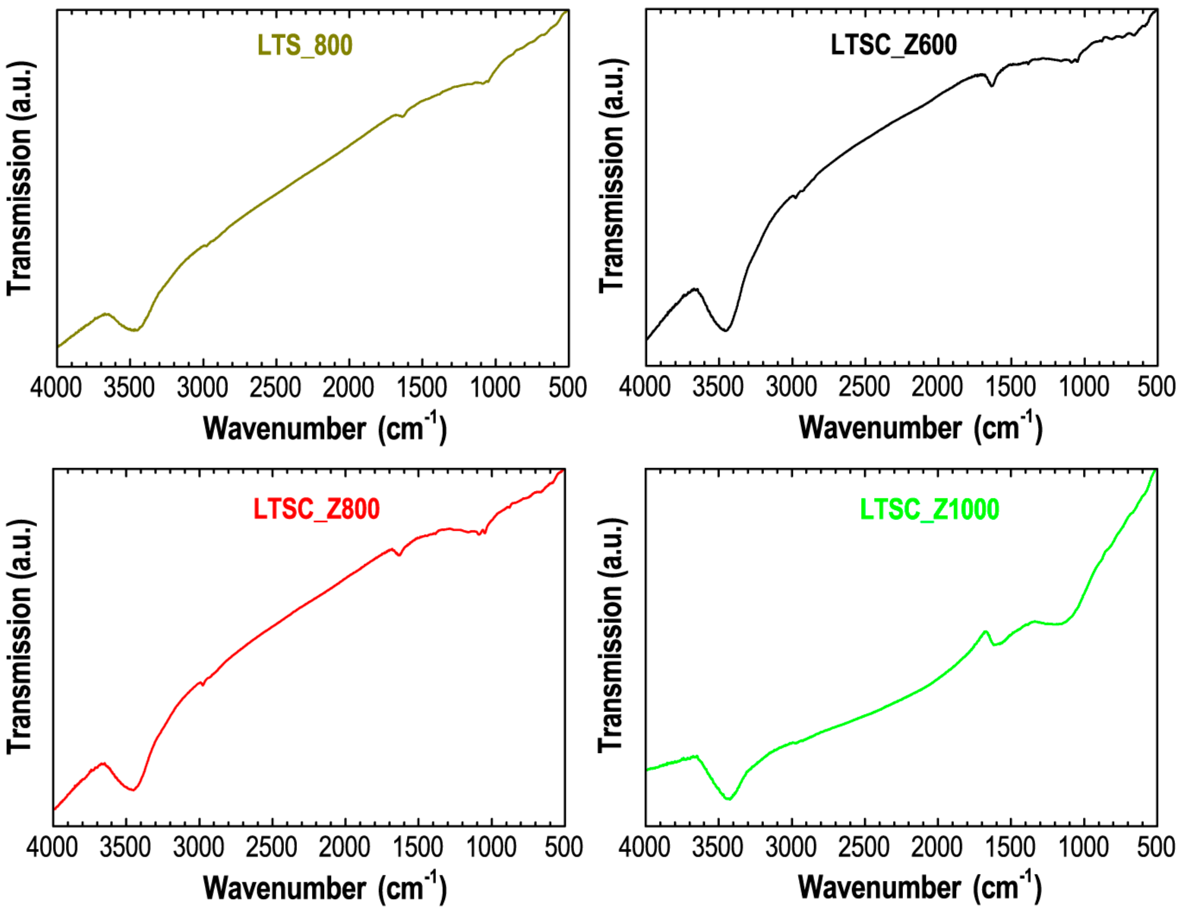

Figure S5. FTIR spectra of Lotus seed-derived carbons LTS_800, LTSC_Z600, LTSC_Z800, and LTSC_Z1000 recorded at 25 °C.
